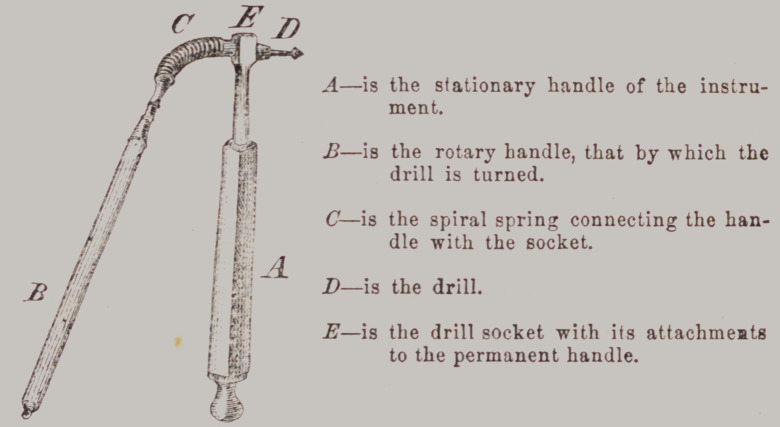# Merry’s Drill Stock

**Published:** 1858-11

**Authors:** 


### MERRY'S DRILL STOCK.

*A*—is the stationary handle of the instrument.

*B*—is the rotary handle, that by which the drill is turned.

*C*—is the spiral spring connecting the handle with the socket.

*D*—is the drill.

*E*—is the drill socket with its attachments to the permanent handle.

This drill stock will be better understood from our engraving, than it can be from any written or verbal description. The operator has as perfect control of the instrument, as he has of the common straight rotary drill. There is no complex machinery by which the drill is worked. It is turned by the fingers. That portion of the instrument which holds the drill being connected with the handle by a spiral spring, the drill can be made to work in any direction, by simply bending the spring. The drills are inserted into a common "taper socket." But the engraving is the best description we can give.—*Dent. Reg.*

A writer in one of our exchange papers says, that coperas, which costs but three cents per pound, is perhaps one of the most efficient and economical disinfecting agents known.

## Figures and Tables

**Figure f1:**